# Enlargement of the Axial Length and Altered Ultrastructural Features of the Sclera in a Mutant Lumican Transgenic Mouse Model

**DOI:** 10.1371/journal.pone.0163165

**Published:** 2016-10-06

**Authors:** Yanzheng Song, Fengju Zhang, Yanyan Zhao, Mingshen Sun, Jun Tao, Yanchuang Liang, Ling Ma, Yanqiu Yu, Jianhua Wang, Junfeng Hao

**Affiliations:** 1 Beijing Tongren Eye Center, Beijing Tongren Hospital, Capital Medical University, Beijing Ophthalmology & Visual Sciences Key Lab, Beijing, China; 2 Department of Pathophysiology, China Medical University, Shenyang, China; 3 Institute of Biophysics, Chinese Academy of Sciences, Beijing, China; University of Hong Kong, HONG KONG

## Abstract

*Lumican (LUM*) is a candidate gene for myopia in the *MYP3* locus. In this study, a mutant *lumican* (L199P) transgenic mouse model was established to investigate the axial length changes and ultrastructural features of the sclera. The mouse model was established by pronuclear microinjection. Transgenic mice and wild-type B6 mice were killed at eight weeks of age. Gene expression levels of *LUM* and *collagen type I (COL1)* in the sclera were analyzed by quantitative real-time polymerase chain reaction (qPCR), and the protein levels were assessed by Western blot analysis. Ocular axial lengths were measured on the enucleated whole eye under a dissecting microscope. Ultrastructural features of collagen fibrils in the sclera were examined with transmission electron microscopy (TEM). *Lumican* and *collagen type I* were both elevated at the transcriptional and protein levels. The mean axial length of eyes in the transgenic mice was significantly longer than that in the wild-type mice (3,231.0 ± 11.2 μm (transgenic group) vs 3,199.7 ± 11.1 μm (controls), *p*<0.05 =). Some ultrastructural changes were observed in the sclera of the transgenic mice under TEM, such as evident lamellar disorganizations and abnormal inter-fibril spacing. The average collagen fibril diameter was smaller than that in their wild-type counterparts. These results indicate that the ectopic mutant *lumican (L199P)* may induce enlargement of axial lengths and abnormal structures and distributions of collagen fibrils in mouse sclera. This transgenic mouse model can be used for the mechanistic study of myopia.

## Introduction

Myopia, especially high myopia (<-6.0 D), is a highly prevalent, complex ocular phenotype and public health problem worldwide[1]. Some etiological studies show that genetic factors play an important role in the development of high myopia[1, 2]. Some genes have been identified as candidate genes of high myopia[3–11], although it is still uncertain which genes are specifically responsible for myopia.

A series of changes has been observed in myopic human eyes (excessive axial elongation, thinner sclera, and even posterior staphyloma in some high myopic eyes) [12],[13], especially in the sclera, which consists of fibroblasts and a lamellar structure of collagen fibers and is considered an important target tissue during the development of myopia. However, it is not easy to obtain myopic human scleral specimens without pathological changes due to other ocular diseases, as the diagnosis of myopia does not allow for a tissue biopsy. Some findings regarding myopic structural changes were based on animals with experimentally induced myopia, but the genetic effects associated with myopia cannot be mimicked in this manner. Thus, the exact changes that occur due to the mutations detected in myopia are unknown.

*Lumican (LUM)* is one of the genes reported to be associated with myopia in many studies. It is located on chromosome 12q22-23 *(MYP3)* [14, 15]. Lumican is expressed in the tendon, cartilage, skin, and ocular sclera[16, 17]. Majava’s DNA analyses[18] revealed a *c*.*596T>C* change in *LUM*, with a CTG codon for leucine 199 converted to CCG for proline (*L199P*) in an English myopic patient. This conversion was speculated to impair protein function.

The purpose of this study was to establish a mutant *lumican (L199P)* transgenic mouse model and to investigate the possible resulting ocular changes, especially in the sclera and its ultrastructure.

## Materials and Methods

### The Establishment of Transgenic Mice

#### Plasmid construction

To construct the destination vector, the mutation of human *LUM* was created by site-directed mutagenesis (*c*.*596T > C*, *L199P*), and the transgene of mutant *LUM (L199P)* was synthesized and cloned with a specific tag incorporated into the target gene sequences to obtain *LUM/flag*. In addition, the humanized renilla green fluorescent protein (*hrGFP*) was introduced into our destination vector as a reporter of expression. The junctions of promoter *elongation factor 1-alpha* (*EF1A*) and the gene fragments *LUM/Flag* and *IRES/hrGFP* were created by gateway cloning (Invitrogen, Life Technologies Australia Pty Ltd, Mulgrave, Victoria, Australia) according to the standard protocol from the gateway technology manual. The entire gene of the plasmid *pEF1A-LUM/Flag-IRES-hrGFP* was sequenced prior to microinjection.

#### Generation of the mutant lumican transgenic mouse model

All the animals used in this study were housed in a specific–pathogen–free (SPF) condition in 12-hour light/dark cycles, with the light level at approximately 250 lux. The temperature of the housing room was maintained at 21°C and the relative humidity was 45~55%; food and water were available ad libitum. Mice were health checked daily throughout the experiment. The cages were changed, and the body weight of each mouse was measured weekly. If mice lost weight (~20% reduction in body weight), stopped eating or drinking, displayed reduced physical activity, or lost hair, they were removed from experimental conditions and euthanized via cervical dislocation. No animals became ill or died prior to the experimental endpoint. All procedures were conducted according to the Association for Research in Vision and Ophthalmology (ARVO) Statement for the care and use of Animals in Ophthalmic and Vision Research and were approved by the Animal Ethical Committee of Capital Medical University, China. The approval number was AEEI-2015-169. Fertilized oocytes were obtained from C57BL/6 (B6) × DBA/2 hybrid female mice. The transgene was purified and microinjected into the zygote male pronuclei of one-cell stage embryos. Then, the living zygote cells were transplanted into the oviduct of pseudopregnant female ICR-strain mice.

Genotypes of the progeny mice were identified by tail biopsies and subsequent polymerase chain reaction (PCR) analyses. Genomic DNA was extracted from the mouse tail, and the primers were designed for the amplification of *hrGFP* to yield a 262-bp fragment using Primer premier 5.0 software (available on http://www.premierbiosoft.com/index.html). The primer sequences were *F-**CCGAGGACATCAGCGACTT* and *R-**AGGATCACCTGGCCCACC*. PCR reactions were carried out according to instructions from the manufacturer (TaKaRa, TaKaRa Biotechnology (Dalian) Co., Ltd, Dalian, China). Then, the reaction mixtures were removed and analyzed on a 2% agarose gel with ethidium bromide staining. The 262-bp DNA band was amplified from the transgenic mouse genomic DNA and visualized by ultraviolet transillumination.

The genomic DNA of the transgenic mice was Southern blotted for further analysis. Genomic DNA was digested with restriction enzymes *EcoRI* and *BglII*. DNA fragments were separated by a 1.0% agarose gel, transferred onto a nylon membrane, and cross-linked to the membrane by ultraviolet light. The hybridization was performed with digoxigenin (DIG)-labeled probes (Mylab DIGD-110, Mylab, Beijing, China) overnight following the manufacturer’s instructions. The primers used for probe labelling were *F-**ATGAGTCTAAGTGCATTTACTCTCT* and *R-**TTACTTGTCATCGTCGTCCTTGT*.

The confirmed progeny mice carrying the transgene were backcrossed 9–10 generations to B6 mice to establish lines from each founder and to produce the heterozygotes used in the following experiments. The genotype of each progeny was confirmed for the presence of the mutant LUM transgene by PCR analyses. The primer sequences were the same as those used in the Southern blot analysis. PCR reactions were carried out according to the same instructions described above. The 958-bp DNA band was amplified from the transgenic mouse genomic DNA and visualized by ultraviolet transillumination. Wild-type B6 mice were used as controls in this study.

Measurements of both the transgenic mice and the wild-type mice used in this study were obtained at eight weeks of age. While the animals were under deep anesthesia (100 mg/kg sodium pentobarbital), both eyeballs were enucleated, and the extraocular muscles and other adherent tissues outside the eyeballs were removed quickly (within 6 minutes of enucleation) under a dissecting microscope (Olympus SZX12, Olympus, Tokyo, Japan) with a halogen cold light source (Schott Illuminator KL1500 LCD, Schott Lighting and Imaging, Southbridge, MA) and placed in ice-cold phosphate-buffered saline (PBS) before subsequent investigations. After the sample collection of the eyeballs, the mice were euthanized with an overdose of sodium pentobarbital (IP, 200 mg/kg).

### Gene Expression Analyses

*LUM* and *collagen type I (COL1)* gene expression levels in the sclera of transgenic (n = 10) and wild-type (n = 10) mice were analyzed by quantitative real-time PCR (qPCR). The corneas were removed with scissors, with cuts made close to the limbus. After removing the lens and vitreous, the inner and outer scleral surfaces were scraped to remove the retina, the choroid, and any remaining extraocular tissue. The optic nerve head was also removed from the posterior pole of the eye. The scleral samples were the only tissues collected from these eyeballs and were homogenized in a freezer mill. After homogenization, total RNA from the scleral samples was extracted using the RNeasy RNA extraction kit (Qiagen, Valencia, CA) according to the manufacturer’s protocol. DNase I was used to remove any potential genomic DNA contamination. The RNA was quantified and checked for purity and condition by spectrophotometry and gel electrophoresis. To generate cDNA, 1 μg of total RNA was reverse-transcribed using murine leukemia virus reverse transcriptase (Invitrogen Life Technologies, Gaithersburg, MD) with primer mix, according to the manufacturer’s instructions. The cDNA samples were stored at -20°C until further use.

qPCR was performed using SYBR Green I reagent (Qiagen, Valencia, CA) on the ABI 7500 system (Applied Biosystems, Darmstadt, Germany). The primer sequences and product sizes are presented in Table 1. For each sample, PCR amplification of the house-keeping gene, β-actin, was performed for normalization. Melting curves were used to ensure the purity of the PCR products. The semi-quantitative comparison between samples was performed using the ΔΔC_T_ method[19] as follows: the data were normalized by subtracting the differences in the threshold cycles (C_T_) between the C_T_ of the gene of interest and that of β-actin (gene of interest C_T_-β-actin C_T_ = ΔC_T_) for each sample. The relative values of the expression levels of these samples were calculated as 2^-ΔCT^. The fold changes of the expression levels between the transgenic group and the wild-type group were calculated as 2^(-transgenic mice group ΔCT + control group ΔCT)^. The PCR assays of each sample were performed in triplicate to improve the accuracy of the estimates.

**Table 1:** Quantitative real-time PCR primer sequences and PCR product sizes.

Gene	Primer sequence	Product size (bp)
*COL1*	*5’-GACGCCATCAAGGTCTACTG-3’*	154
	*5’-ACGGGAATCCATCGGTCA-3’*	
*LUM*	*5’-CGAAAGCAGTGTCAAGACAGTAA-3’*	205
	*5’-CACAGTACATGGCACTTGGGTAG-3’*	
*β-actin*	*5’-TTTTCCAGCCTTCCTTCTTGGGTAT-3’*	111
	*5’-CTGTGTTGGCATAGAGGTCTTTACG-3’*	

### Western Blot Analyses

Lumican and collagen type I in the sclera were analyzed at the protein level by Western blotting. The scleral samples were collected from both animal groups as described above for gene expression analyses. The scleral tissue was homogenized and solubilized in ice-cold PBS containing PMSF protease inhibitor (Abcam/ab141032, Abcam Inc., Cambridge, MA). The total protein concentration was determined using a BCA Protein Assay Kit (cat. no. 23250, Thermo fisher Scientific, Waltham, MA). Polyacrylamide gel was prepared for sodium dodecyl sulfate-polyacrylamide gel electrophoresis (SDS-PAGE). The samples were loaded, and the gels were electroblotted at 70 V. The separated proteins were transferred to a polyvinylidene difluoride (PVDF) membrane. The primary antibodies used in this study were a monoclonal antibody directed against lumican (Abcam/ab168348, Abcam Inc., Cambridge, MA) and anti-COL1A2 (Abcam/ab96723, Abcam Inc., Cambridge, MA). In addition, the protein GAPDH (Abcam/ab8245, Abcam Inc.) was examined as a control, and the anti-FLAG M2 antibody (Sigma/F3165, Sigma-Aldrich Co. LLC., St. Louis, MO) was used to verify the transgenic mice. Goat anti-rabbit IgG-HRP (Abcam/ab6721, Abcam Inc.) was used as a secondary antibody. The quantification of lumican, COL1A2, and GAPDH was determined by analyzing band density using image J (National Institutes of Health; Bethesda, MD). The experiments were repeated three times with similar results.

### Axial Length Measurements

Twenty mutant lumican transgenic female mice and twenty-two wild-type age-matched female mice were examined. The enucleated eyeballs were kept in ice-cold PBS (PH 7.4) to prevent drying. The eyes were viewed with a 20× objective under a dissecting microscope. Three images of each eyeball were obtained by the SPOT camera within 5 minutes and projected onto a computer screen. The axial length measurements were made with Spot Advanced software (Diagnostic instruments, Sterling Height, MI). A straight line was drawn by a masked observer from the center of the cornea to the base of the optic nerve on the image, allowing the axial length data to be read by the software’s calibrated automated measuring tool with an accuracy of 1 μm. Every image was measured at least five times, and the average of three images was used as the final value for each eyeball.

### Transmission Electron Microscopy Examinations

Five mice from each group underwent TEM examinations. The eyes were enucleated and fixed in 3% glutaraldehyde for 15 minutes, during which time the corneas were removed. The sclera was further fixed at 4°C for 24–48 hours and then placed in 1% osmium. After dehydration in a graded ethanol series, the tissues were embedded in an epoxy resin mixture at 60°C for 48 hours. The optic nerves were used as a marker to assist with orientation while embedding tissues. Thick sections (1 μm) were cut from the posterior sclera and stained with methylene blue for the selection of specific regions for further study, and thin sections (100 nm) were taken from the peripapillary sclera and placed on copper mesh grids for TEM examinations at 75 kV (JEM-1200EX, JEOL, Tokyo, Japan).

Digital images were obtained in at least five sections of the posterior sclera. A low-magnification image (1,000×) of each section was obtained initially to observe the overall scleral lamellae in an entire field, followed by a series of images (3,000×–8,000×) to assess the fibers of the scleral lamellae in detail. High-magnification images (8,000×) were amplified to 40,000× and saved using Adobe Photoshop software. Then, the collagen fibril diameter was measured in the peripapillary region in 10 to 18 fields of each sclera. The circle-shaped fibrils, which were oriented in a cross-section, were measured by image analysis software (Leica Qwin Standard V2.8 image analysis software, Leica, Cambridge, UK). Using a line tool, a masked observer individually highlighted the rims of the circle-shaped fibrils. Because the rims were not perfect standard circular shapes, the diameters of the collagen fibrils were calculated from the fibril areas, which were obtained from the software in an automated manner. According to the distribution of the cross-sectional collagen fibrils in the visual field of the microscope, approximately 60 to 300 fibril diameters from a single field were calculated for the purpose of statistical analysis.

### Statistical Analysis

Student’s t-test was used to determine the statistical significance levels of the axial lengths as well as the gene and protein expression levels of Lumican and collagen type I. Because the fibril diameter data from the transgenic mice were skewed, quartiles were used to describe the frequencies, and the nonparametric Mann-Whitney U test was used to analyze the difference between the transgenic and wild-type mice. These statistical analyses were performed using SPSS software, version 17.0 (SPSS Science, Chicago, IL, USA).

## Results

### Establishment and Characterization of Transgenic Mice

**Fig 1:**
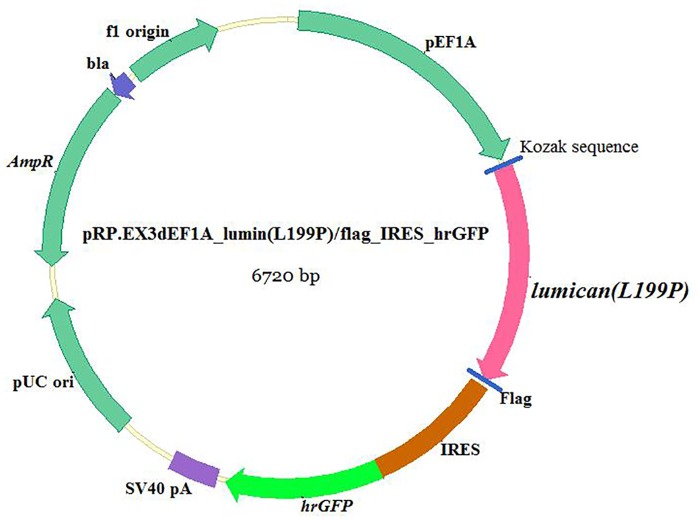
Diagram of plasmid structure carrying the mutant *LUM (L199P)*. The *EF1A* was introduced into the destination vector as a promoter, and the *Flag* and *hrGFP* tags were introduced as reporters of expression.

**Fig 2:**
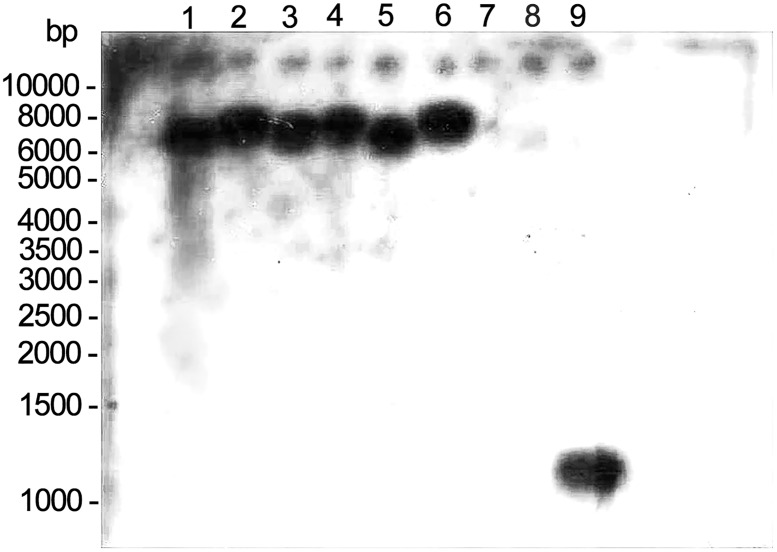
Southern blot hybridization results. The genomic DNA extracted from six transgenic mice and one wild-type mouse was digested with EcoR I and Bgl II and hybridized using a DIGD-110-labeled probe. Lane 1 to lane 6, EcoR I- and Bgl II-digested DNA from six mouse tails (No. 9, No. 19, No. 20, No. 22, No. 24, and No. 31). Lane 7, genomic DNA fragments from the wild-type mouse as a negative control. Lane 8 to lane 9, PCR products of transgene *LUM* as positive controls.

**Fig 3:**
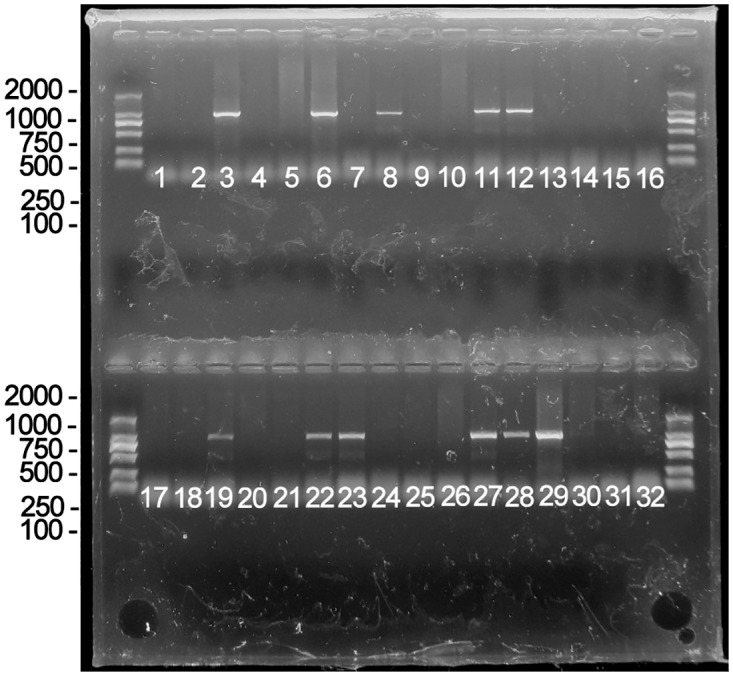
Agarose gel electrophoresis of PCR-amplified 958-bp-fragment mutant lumican from the transgenic mouse tail tissue. Thirty-two F1 mice were examined by PCR. As the results indicated, No. F1-3, No. F1-6, No. F1-8, No. F1-11, No. F1-12, No. F1-19, No. F1-22, No. F1-23, No. F1-27, No. F1-28, and No. F1-29 were transgenic mice, while the others did not carry the transgene.

The characterization of the transgenic construct carrying the target gene is shown in Fig 1. The linearized, gel-purified transgenes were microinjected into 202 fertilized one-cell stage embryos. From these microinjections, 162 embryos survived and were transplanted into the oviducts of accepter female ICR mice. A total of forty mice were born and were identified by a tail biopsy. The exogenous gene fragment hrGFP (262 bp) from the mouse tail was detected by PCR, and 6 of the 40 mice (No. 9, No. 19, No. 20, No. 22, No. 24, and No. 31) were found to have the transgene. To confirm this result, genomic DNA extracted from these six transgenic mice was further analyzed by Southern blot analysis, and the results are shown in Fig 2. The genotypes of heterozygotes used in the following experiments were also identified by PCR analyses (Fig 3).

### Increased Gene Expression Levels of *LUM* and *COL1*

**Fig 4:**
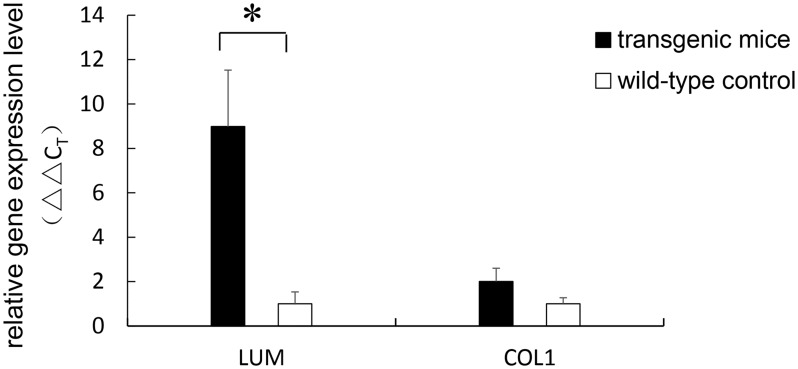
Total scleral gene expression levels of *LUM* and *COL1*. The relative gene expression levels were calculated using the ΔΔC_T_ method. The expression level of *LUM* was found to be 8.85-fold increased relative to the control level (p = 0.023). The expression level of *COL1* was 2.01-fold greater in transgenic mice; however, the difference was not statistically significant (p = 0.086). Error bar = 1 SEM. * *p*<0.05.

Analyses of the gene expression of *LUM*, normalized to the expression of the house-keeping gene *β-actin*, showed a significant increase in the expression throughout the entire sclera of the transgenic mice compared with that of the wild-type mice (8.85-fold, p = 0.023). The expression level of *COL1* was increased 2.01-fold in the transgenic mice compared with the wild-type mice, although the increase was not statistically significant (p = 0.086). The relative scleral gene expression levels of *LUM* and *COL1* are shown in Fig 4.

### Increased Protein Levels of Lumican and Collagen type I

**Fig 5:**
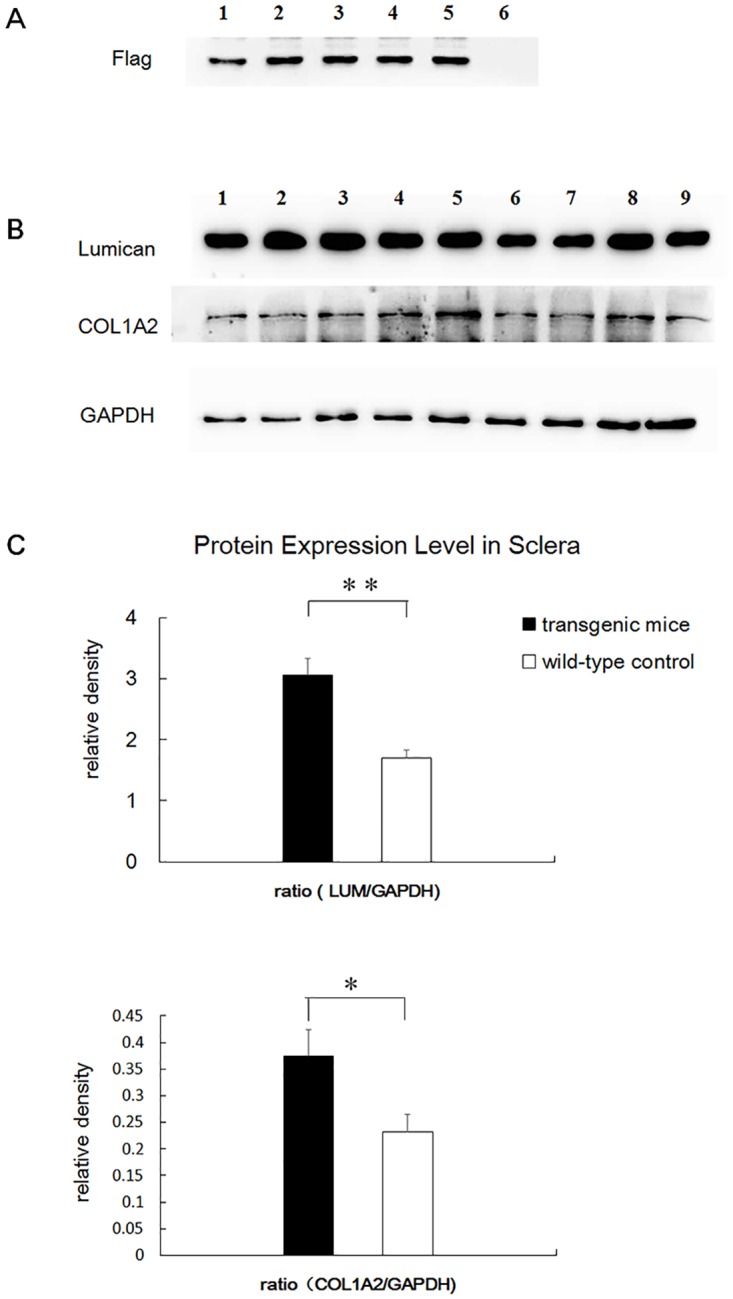
Analysis of protein expression level in the sclera. A. The flag protein was expressed in transgenic mice (lane 1 to lane 5), whereas no corresponding band was found in the wild-type mice (lane 6). B. Lumican and collagen type I were both overexpressed at the protein level in the transgenic sclera. GAPDH was used as a control. Lane 1 to lane 5, transgenic mice. Lane 6 to lane 9, wild-type mice. C. The expression level of lumican and collagen type I was significantly increased. Error bar = 1 SEM. * *p*<0.05, ** *p*<0.01.

As shown in Fig 5A, the flag protein was expressed in the transgenic mice, whereas no corresponding band was found in the wild-type mice. Lumican and collagen type I were both overexpressed at the protein level in the transgenic sclera (Fig 5B). To quantify the expression levels of the lumican and collagen type I genes, the band density was analyzed. Relative density was calculated and normalized using GAPDH as a control (Fig 5C). The expression level of lumican was calculated as a ratio (LUM/GAPDH) and was 3.053 ± 0.275 (mean ± SEM, n = 5) in the transgenic mice, which was significantly greater than that in the wild-type mice (1.700 ± 0.126, n = 4, p = 0.005). The expression level of collagen type I was also significantly increased in the transgenic mice. The ratio (COL1A2/GAPDH) was 0.375 ± 0.049 in the transgenic mice and 0.232 ± 0.033 in the wild-type mice (p = 0.047).

### Increased Axial Length in Transgenic Mice

**Fig 6:**
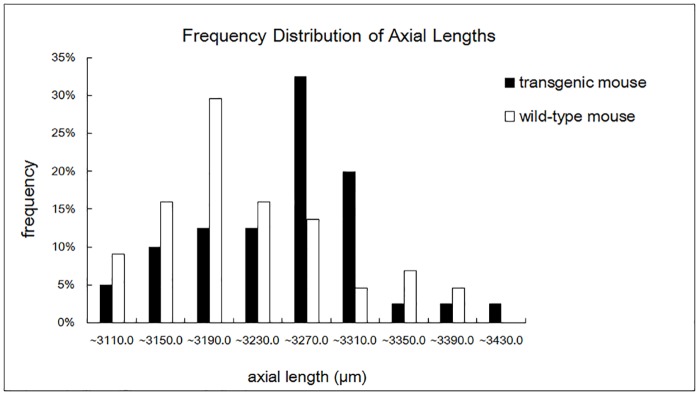
The frequency distribution of the axial length in transgenic and wild-type mice.

**Fig 7:**
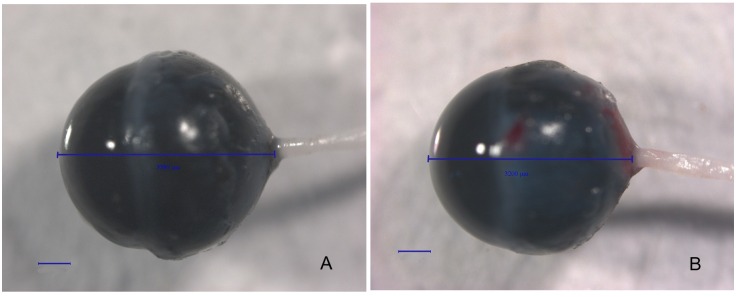
Photographs of the eyeballs of mutant *lumican* transgenic and wild-type mice. These eyeballs were observed with a 20× objective. The axial lengths are shown with a line drawn from the anterior surface of the cornea to the base of the optic nerve. Bar, 500 μm. A. transgenic mouse. B. A wild-type mouse.

Both the mutant *lumican* transgenic mice and the wild-type mice were killed at the 8^th^ postnatal week. The eyeballs were enucleated, viewed, and photographed with a 20X objective under a dissecting microscope. The axial lengths of these age-matched mice were compared. The mean axial length in the transgenic mice was 3,231.0 ± 11.2 μm (mean ± SEM, n = 40 eyes from 20 mice), which was significantly greater than that in the control group (3,199.7 ± 11.1 μm, n = 44 eyes from 22 mice, *p* < 0.05). The frequency distribution of the axial length in the transgenic and wild-type mice is shown in Fig 6. A photograph of the transgenic eyeball is in Fig 7A compared with a wild-type eyeball (Fig 7B). The axial length of the transgenic mouse showed in Fig 7 is 3,389 μm, while the axial length of the wild-type mouse is 3,200 μm.

### Changes of the scleral lamellar structure in transgenic mice

**Fig 8:**
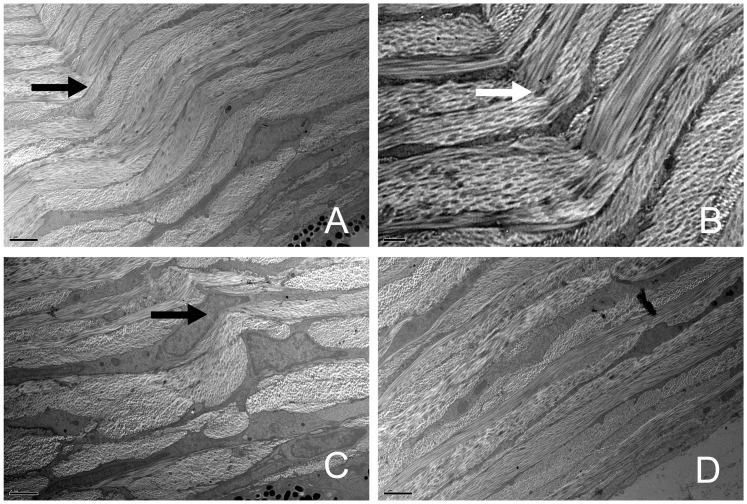
TEM images of the scleral lamellae. Four types of lamellae were seen in the low-magnification images. A. Some of the cross-sectional lamellae, longitudinal lamellae, and oblique lamellae were twisted together (black arrow, 1,000×). B. Part of Fig 6A shown in detail at 3,000× magnification (white arrow). C. Some scleral fibroblasts (black arrow) within the twisted lamellae exhibited an altered morphology (1,000×). D. The scleral lamellae were regularly arranged in wild-type mice (1,000×). The bar in A, C, and D is 2 μm; and the bar in B is 0.5 μm.

**Fig 9:**
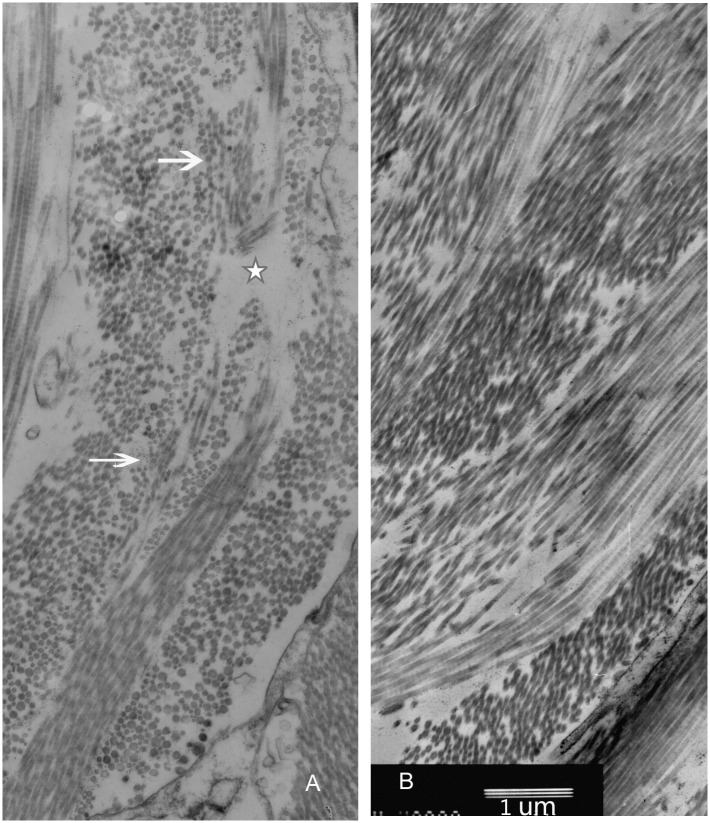
TEM images of collagen fibrils (8,000×). A. Some collagen fibril bundles in the longitudinal lamellae seemed to be “disrupted” in orientation and separated by cross-sectioned fibrils (arrows). There is also evidence of inter-fibril spacing (five-pointed star). B. The arrangement of scleral collagen fibrils in wild-type mice was more regular. Bar, 1 μm.

The sections obtained from the peripapillary sclera were observed. Based on the orientation of collagen fibrils, the lamellae were classified into four types: cross-sectional lamellae (fibrils oriented in the anterior-posterior direction), longitudinal lamellae (parallel to equator), oblique lamellae, and cellular lamellae (consisting of scleral fibroblasts). All four types were observed in both the transgenic and wild-type mice in the low-magnification images (1,000X). In the wild-type mice, the lamellae were regularly arranged. However, in some transgenic mice, the lamellae arrangements were irregular and had distorted directions. Some of the cross-sectional lamellae, longitudinal lamellae, and oblique lamellae were twisted together (Fig 8A), which is shown in detail in Fig 8B (3,000X). The shapes of scleral fibroblasts within the twisted lamellae were also changed (Fig 8C). None of these ultrastructural alterations were found in the wild-type mice (Fig 8D). A detailed evaluation of the organization of the collagen fibrils in the TEM micrographs (8,000X) showed that some collagen fibril bundles in the longitudinal lamellae seemed to be “disrupted” in orientation and separated by cross-sectional fibrils. Additionally, some evident inter-fibril spacing was observed in these “disrupted” lamellae (Fig 9A), while the scleral collagen fibrils observed in the wild-type mice were more regular than those in their transgenic counterparts (Fig 9B).

### The Reduced Collagen Fibril Diameter and the Increased Frequency of Small-diameter Collagen Fibrils

**Fig 10:**
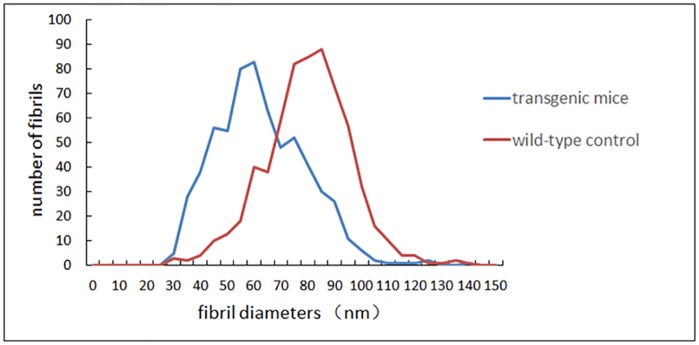
Fibril diameter distributions in transgenic and wild-type mice. Generally, the fibril diameters in the transgenic mice were smaller than those in the wild-type controls.

The ultrastructure of the collagen fibrils throughout the entire lamellae of the peripapillary sclera was photographed and digitized using a transmission electron microscope (40,000X) to measure the diameter. The cross-sectional collagen fibrils were measured and analyzed. Generally, the fibril diameters in the transgenic mice were smaller than those in the wild-type controls. In the transgenic mice, the 25th percentile, 50th percentile (median), and 75th percentile of fibril diameters were 52.78, 63.34, and 76.45 nm, respectively (ranging from 31.83 nm to 145.58 nm), while the 25th, 50th (median), and 75th percentiles of fibril diameters in the wild-type mice group were 72.85, 82.75, and 92.47 nm, respectively (ranging from 32.46 nm to 144.32 nm). There was a significant difference between the two groups (Mann-Whitney U test, *p*<0.001). The distributions of collagen fibril diameters in the transgenic and wild-type mice are shown in Fig 10.

## Discussion

The etiology of myopia involves genetic factors[20]. Genome-wide association studies in large-scale populations have provided great insight into the genetics of human myopia[21–23]. The Consortium for Refractive Error and Myopia (CREAM) and the 23andMe study have identified more than 20 genome-wide loci, which were proximal to genes related to the biological processes of synaptic neurotransmission, ion channel activities, retinoic acid metabolism, scleral extracellular matrix (ECM) remodeling, and even ocular development. Among these loci, several were identified to be associated with the ocular axial length in high myopia[24]. In previous studies, some genetic loci associated with high myopia were mapped to 12q21-q22, including the locus for *LUM*. *LUM* is a candidate gene associated with high myopia, especially in Asian populations[25–28]. In our previous study, we verified that *LUM* is susceptible to pathological myopia in the northern Han ethnic Chinese people[29]. The *L199P* change in *lumican* was speculated to be the cause of high myopia in an individual participant[18], as this amino acid is highly conserved during evolution. High homology is shared among various species.

Lumican is a member of the small leucine-rich repeat protein/proteoglycan *(SLRP)* family, along with opticin, decorin, biglycan, fibromodulin, and proline arginine-rich end leucine-rich repeat protein (PRELP)[30], characterized by a protein core composed of leucine-rich repeats (LRRs). LRRs are short sequence motifs that have been identified in a large number of proteins. The leucine-rich repeats commonly fold together to form a solenoid protein domain, termed the leucine-rich repeat domain. Each repeat unit typically has a β-strand-turn-α-helix structure, and the assembled domain has a horseshoe shape with an interior parallel β-sheet and an exterior array of helices[31, 32]. LRRs are frequently involved in the formation of protein-protein interactions[33]. Collagen is a major constituent in the scleral ECM, and collagen type I accounts for approximately 99% of scleral collagen[34]. Lumican moiety binds collagen fibrils and regulates collagen fibril organization and circumferential growth. It interacts with collagen and regulates collagen fibril diameter and spacing by inhibiting the lateral assembly of collagen molecules from spontaneously forming collagen fibrils. Thus, the production of collagen fibrils is controlled, and the fibril diameter is limited[35]. Lumican contains 11 LRR motifs, and the mutation studied in our research (*L199P*) is located in the region of LRR 6 (amino acids 185–205), which is in the central domain of lumican (Lum 5–7, amino acids 147–220). The central domain of lumican plays an important role in binding to collagen type I. Mutations in this domain impaired the interaction of lumican with collagen[36]. The *L199P* mutation is suspected to affect the tertiary structure of the lumican protein. The misfolded protein may affect the interaction with collagen type I and may contribute to the malfunction of collagen fibrils.

It should be noted that *LUM* expression transcription and protein levels are both higher in transgenic mice than in wild-type mice. Currently, we do not know whether both the mutation and the overexpression of lumican contribute to axial elongation. Interestingly, lumican overexpression in mice does not lead to alterations in the corneal collagen[37]. Because the composition and structure are similar in corneal and scleral ECM, it is speculated that the overexpression of *LUM* in the sclera may not introduce structural changes to the collagen in the sclera. Based on our TEM analyses, the collagen ultrastructure and fibril diameters in lumican transgenic mice are different from those in wild-type control mice. It is therefore speculated that the mutant lumican protein cannot function properly. It may disrupt the formation and arrangement of collagen fibrils, resulting in an increased number of small-diameter collagen fibrils, irregular lamellae, distorted fibril directions, and abnormal inter-fibril spacing.

During ocular development, the regulation of collagen fibrils increases the elasticity and tension of the sclera and controls the axial length and eye shape. The biomechanics of the sclera may be affected by the ultrastructural changes in the collagen fibrils, which is assumed to be a pathological basis of myopia and even some other diseases secondary to high myopia. The ultrastructural anomalies in collagen in our mutant *lumican* transgenic mice may affect the elasticity and tension of the sclera and consequently lead to an elongated ocular axial length.

Our results have also been supported by some lumican-knockdown animal experiments. To better understand the role of lumican in the sclera, some lumican-knockdown animal models have been used in studies of myopia. Lumican-null mouse[38] and lumican-null zebrafish[39] animal models have some abnormalities in the formation of collagen fibrils. The lamellae in the sclera are irregularly arranged, especially in the posterior scleral region, supporting the role of lumican as a regulator of collagen fibril organization. In addition to the ultrastructure changes in collagen fibrils in the sclera, a larger sized eyeball accompanied by a significantly thinner sclera was also noted in the lumican-knockdown zebrafish. The lumican-fibromodulin double-null mouse has an increased axial length, thin sclera, and even retinal detachment[40]. These are all manifestations of high myopia in humans. However, the collagen fibril diameters in these lumican-knockdown animal models are larger than those in the wild-type controls. The diameters of knockdown animal models are altered in an opposite direction compared with that of our transgenic mouse model and human[13] (smaller fibril diameters than those of the wild-type controls). In addition, the lumican-knockdown mouse has a cloudy cornea because of the complete absence of lumican[41]. In conclusion, lumican plays an important role in ocular development. Both knockout and ectopic mutant lumican can affect collagen fibril growth and can consequently impair the ocular tissues. Proper collagen fibril diameters are critical for scleral elasticity and tension.

Based on animal studies[34, 42], it is believed that mammalian scleral remodeling is associated with a general loss of collagen, especially in the posterior sclera. When analyzing our results on the expression levels of collagen type I, instead of decreasing as observed in other studies on myopia, the expression levels of collagen type I increased at both the transcriptional and protein levels. As one of the functions of lumican is collagen fibril binding, it is reasonable to speculate that the overexpression of collagen type I is due to the overexpressed lumican protein.

This study suggests that *LUM* may be a key gene associated with myopia. The mutation of *lumican (L199P)* can cause myopic changes with abnormal collagen fibril structures and distributions in transgenic mice. However, high myopia is a complex eye disease. The occurrence of this disease involves multiple genetic factors, environmental factors, and even a combination of genetic and environmental factors[43–45]. It is complicated and difficult to identify susceptibility factors. To date, few positive results have been convincingly replicated in experimental myopia animal models. Therefore, hopefully, the mutant lumican transgenic mouse can play a role in the investigation of the pathogenesis of high myopia and may elucidate factors related to the scleral changes.
